# A novel influenza vector-based vaccine expressing ESAT-6 and TB10.4 confers immunity and protection against *Bovine tuberculosis* in guinea pigs and calves

**DOI:** 10.14202/vetworld.2025.2573-2589

**Published:** 2025-09-06

**Authors:** Ainur Nurpeisova, Zhandos Abay, Markhabat Kassenov, Nazym Syrym, Sandugash Sadikaliyeva, Bolat Yespembetov, Kuanysh Jekebekov, Ruslan Abitayev, Syrym Kopeyev, Aisha Issabek, Yeraly Shayakhmetov, Elina Kalimolda, Zharkinay Absatova, Sabina Moldagulova, Makhpal Sarmykova, Han Sang Yoo, Berik Khairullin, Kainar Barakbayev, Yerbol Bulatov, Sergazy Nurabayev, Kunsulu Zakarya, Aslan Kerimbayev, Kamshat Shorayeva

**Affiliations:** 1 Laboratory for Control of Technologies and Biopreparations, Research Institute for Biological Safety Problems LLP, Guardeyskiy uts 080409, Kazakhstan; 2 Laboratory of Infectious Diseases, College of Veterinary Medicine, Seoul National University, Seoul 08826, Republic of Korea; 3 MVA Group Scientific-Research Production Center Ltd., Almaty 050046, Kazakhstan

**Keywords:** *Bovine tuberculosis*, calves, ESAT-6, guinea pigs, interferon-gamma, influenza vector, *Mycobacterium bovis*, protective efficacy, TB10.4, vaccine

## Abstract

**Background and Aim:**

*Bovine tuberculosis* (bTB), caused by *Mycobacterium bovis*, remains a significant zoonotic and economic threat globally. Despite the long-standing use of the Bacillus Calmette-Guérin (BCG) vaccine, its inconsistent efficacy and interference with surveillance tests underscore the need for alternative approaches. This study evaluated the safety, immunogenicity, and protective efficacy of a novel influenza vector-based vaccine expressing *M. bovis* antigens ESAT-6 and TB10.4, formulated with or without an adjuvant.

**Materials and Methods:**

Recombinant influenza A viruses expressing ESAT-6 and TB10.4 were constructed using reverse genetics and incorporated into vaccine formulations. Guinea pigs and calves were immunized with adjuvanted and non-adjuvanted formulations, followed by challenge with a virulent *M. bovis* strain. Safety was assessed through clinical observation and histopathology. Immune responses were monitored using interferon-gamma (IFNγ) enzyme-linked immunosorbent assay, and protection was evaluated through organ damage indices, bacterial load, and survival rates over a 12-month period.

**Results:**

Both formulations were safe and well-tolerated in guinea pigs and calves, with no adverse clinical signs. The non-adjuvanted vaccine induced the highest and most sustained IFNγ response, peaking between 2 and 5 months post-vaccination. In guinea pigs, the protection index reached +0.60 lg in the non-adjuvanted group versus +0.2 lg in the adjuvanted group. In calves, lung bacterial load was reduced to 1.83–1.93 lg colony-forming unit (CFU) in vaccinated animals compared with 5.8 lg CFU in unvaccinated controls. Histopathological examination confirmed minimal tissue damage in the vaccinated groups. Both vaccine formulations demonstrated protective efficacy equivalent to or better than BCG, with the non-adjuvanted version showing superior performance.

**Conclusion:**

This novel influenza vector-based vaccine expressing ESAT-6 and TB10.4 antigens elicits strong, long-lasting cellular immunity and provides significant protection against *M. bovis* infection in guinea pigs and calves. The adjuvant-free formulation demonstrated higher immunogenicity, simplified production, and minimal adverse reactions, positioning it as a promising alternative to BCG for bTB control in livestock.

## INTRODUCTION

Tuberculosis is an infectious disease caused by *Mycobacterium tuberculosis* [1]. *Bovine tuberculosis* (bTB), caused by *Mycobacterium bovis*, is a serious re-emerging disease that affects both animals (farm and wild) and humans [2]. bTB can affect multiple organs, including the lungs, liver, intestines, lymph nodes, kidneys, bones, joints, reproductive system, and mammary glands, with the latter being particularly significant as contaminated milk may serve as a source of zoonotic transmission [3–6].

Many industrialized nations implement preventative measures, such as regular tuberculin testing and culling of infected animals to ensure the proper growth and development of farm animals. These measures have significantly contributed to bTB reduction and eradication in certain regions [7–9]. However, such strategies may not be practical or sustainable in endemic regions, where bTB continues to pose major public health and economic challenges.

Vaccination remains a key tool for halting the spread of infections. In recent decades, immunization with classical inactivated and live vaccines has allowed humanity to control numerous infections and significantly reduce their burden. However, many bacterial pathogens remain for which classical approaches are insufficient. This is often due to the lack of safety associated with classical vaccines, their narrow specificity, or the high variability of pathogen antigenic properties that enable evasion of host immune defenses [10].

Despite the historical success of the Bacillus Calmette–Guérin (BCG) vaccine in reducing tuberculosis incidence in humans, its efficacy in cattle remains variable and often insufficient. BCG does not consistently prevent bovine infection or transmission and can interfere with routine tuberculin-based surveillance programs. These limitations hinder large-scale implementation in livestock and highlight the need for alternative vaccines that are both effective and compatible with bTB control strategies.

Developing an effective bTB vaccine presents several challenges. These include the complex biology of *M. bovis*, variability in immune responses among cattle breeds, the need for efficient delivery systems, and rigorous regulatory approval processes. Moreover, ensuring vaccine safety, efficacy, and cost-effectiveness for large-scale implementation remains a critical priority. Although several vaccine candidates have demonstrated promise in pre-clinical studies, their efficacy in reducing disease severity or transmission has been variable [10].

Viral vectors are a promising strategy for targeted delivery and effective antigen presentation to the recipient’s immune system. Several criteria determine the suitability of a viral vector: safety, absence of pre-existing immunity to the vector, ability to overcome vector immunity during initial and repeated immunizations, and scalability of production [11].

Viral vectors, such as those based on influenza A virus, offer several advantages: native antigen expression, immune-inductive site targeting, and scalable production [10]. These features make them attractive platforms for veterinary vaccines and were key considerations in the design of our candidate [11].

The antigens ESAT-6 and TB10.4 were selected due to their immunogenicity and relevance in the pathogenesis of tuberculosis. These proteins are expressed during the key phases of *M. bovis* infection and contain epitopes recognized by both CD4+ and CD8+ T-cells, supporting robust cellular responses [12]. Moreover, their exclusion from BCG allows these antigens to serve as differentiating markers for differentiating infected from vaccinated animals (DIVA)-compatible strategies.

Despite the global recognition of bTB as a critical zoonotic and economic concern, the development of effective and practical vaccination strategies remains limited. The BCG vaccine, although extensively studied, presents several shortcomings when applied to livestock: inconsistent protective efficacy across cattle breeds, potential interference with tuberculin-based diagnostic tests, and the absence of certain immunodominant antigens such as ESAT-6 and TB10.4. These limitations compromise the feasibility of implementing BCG in large-scale eradication programs and underline the urgent need for next-generation vaccines that are both immunologically robust and compatible with disease surveillance systems. While viral vectors have shown potential as platforms for antigen delivery in human and veterinary vaccines, few studies have explored influenza virus-based vectors for *M. bovis* antigen expression in livestock. Furthermore, comparative assessments of adjuvanted and non-adjuvanted formulations in terms of long-term immune protection, safety, and field applicability in target species are largely lacking. Therefore, there exists a significant research gap in evaluating influenza-based vector vaccines expressing DIVA-compatible antigens for bTB control in animal models relevant to veterinary practice.

This study aims to develop and evaluate a novel influenza vector-based vaccine expressing the *M. bovis* antigens ESAT-6 and TB10.4, delivered through the non-structural protein 1 (NS1) region of the influenza A virus genome. Specifically, the objectives are to (1) assess the safety profile of adjuvanted and non-adjuvanted vaccine formulations in guinea pigs and calves; (2) evaluate the immunogenicity of the formulations by quantifying interferon-gamma (IFNγ) production over a 12-month period post-vaccination; (3) determine the protective efficacy of the candidate vaccines through challenge studies using a virulent *M. bovis* strain; and (4) compare the performance of the novel vaccine candidates against the traditional BCG vaccine in terms of immune persistence, bacterial load reduction, and histopathological outcomes. By addressing the need for safer, DIVA-compatible, and scalable alternatives to BCG, this study provides foundational evidence for the application of influenza-based vectors in veterinary tuberculosis vaccine development.

## MATERIALS AND METHODS

### Ethical approval

All procedures complied with national and international ethical guidelines for animal research, including the UK Animals (Scientific Procedures) Act 1986 and EU Directive 2010/63/EU. The Ethics Committee for Animal Experiments of the Research Institute for Biological Safety Problems (RIBSP) Science Committee reviewed and approved the experimental protocol (Protocol #10, dated September 28, 2020). All laboratory experiments involving live cells were conducted in a BSL-3 facility. Animal experimentation was conducted in a BSL-2 facility.

### Study period and location

The study was conducted from September 2021 to December 2023 at the Research Institute for Biological Safety Problems (RIBSP), Zhambyl region, Kazakhstan.

### Recombinant influenza vectors expressing *M. bovis* ESAT-6 and TB10.4

We generated recombinant influenza viruses expressing *M. bovis* antigens ESAT-6 and TB10.4 using a standard reverse genetic system [10, 12]. The *NS1* gene was modified to include the antigen sequences. The recombinant viruses were rescued in Vero cells and propagated in 10–11-day-old embryonated chicken eggs at 37°C for 48 h [10].

### Preparation of the vaccine

Candidate vaccine formulations were prepared using 10–11-day-old embryonated chicken eggs free from chicken embryo-specific pathogens. The collected allantoic fluid was purified through multiple clarification steps and mixed in a 1:1 ratio with a sterile stabilizing medium containing 12% peptone and 6% sucrose, yielding final concentrations of 6% and 3%, respectively.

In this study, we tested three experimental series of each candidate vaccine: the vector vaccine for tuberculosis containing 15% Montanide Gel adjuvant and the vector vaccine without an adjuvant. We also included a group of animals immunized with saline and a naive group as controls.

### Bacterial strain

The virulent 0078-*M. bovis*-8/RIBSP strain used in our study was obtained from the Microbiology Laboratory collection of the Research Institute for Biological Safety Problems, Kazakhstan. The bacterial cells were cultured on solid egg-based Lowenstein-Jensen medium at 37°C ± 0.5°C.

### Animals

The study included 64 guinea pigs, each weighing 250–300 g and aged 1.5–2 months, and 80 calves aged 6 months and weighing 150–200 kg, all obtained from tuberculosis-free farms.

Before the experiments, all animals were quarantined for 1 month in the experimental animal department. Before beginning the study, animals were tested for specific immune responses to tuberculin using the DIATUB PPD for mammals (TOO NPP “Antigen”, Kazakhstan) according to the manufacturer’s instructions. During the immunization phase, animals were housed in the vivarium of the RIBSP. For subsequent infection experiments with the virulent strain, animals were transferred to a BSL-2 laboratory.

Throughout the entire study, trained personnel closely monitored the animals’ welfare. All animals were observed daily for any signs of distress, abnormal behavior, changes in appetite, or clinical symptoms. Housing, environmental conditions, and access to food and water were maintained according to international animal welfare guidelines.

### Safety and protective effectiveness assessment in guinea pigs

**Figure 1:**
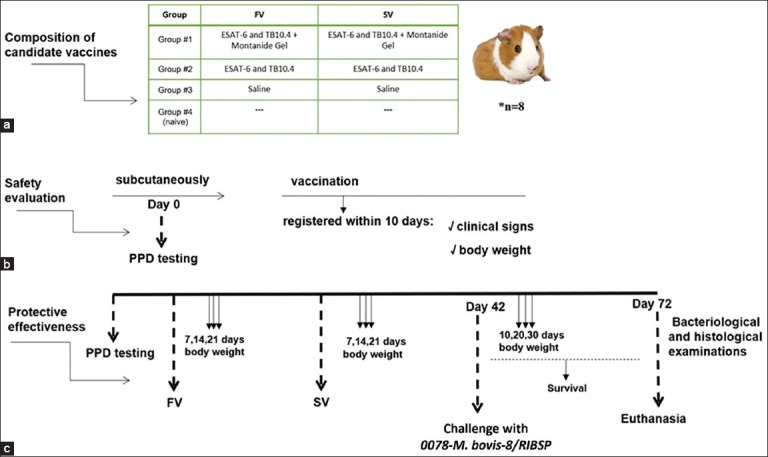
Study design. (a) Composition of candidate vaccines. (b) Assessment of the safety of candidate vaccines based on clinical signs and body weight. (c) Experiment flowchart: assessment of the protective effectiveness of candidate vaccines by challenging guinea pigs with *the 0078-Mycobacterium bovis-8*/RIBSP virulent strain. FV = First vaccination, SV = Second vaccination (revaccination).

To assess safety, 32 guinea pigs were divided into four groups of 8 animals each (n = 8) and immunized with a single 1 mL dose (lg 6.25 egg infective dose (EID) 50/mL) subcutaneously (Figure 1a). During the 10-day observation period, the animals’ general condition was monitored, including food and water intake, skin and mucous membrane condition, coat appearance, behavioral responses, motor activity, and changes in body weight. Body weight changes were recorded throughout the experiment (Figure 1b).

To evaluate protective efficacy, another 32 guinea pigs were randomized into four groups of 8 animals each (n = 8). These animals received two subcutaneous immunizations spaced 21 days apart and were challenged on day 42 with the virulent *M. bovis* strain 0078-*M. bovis*-8/RIBSP at a dose of 1 × 10^6^ CFU per animal in 1.0 mL of saline solution (0.9% NaCl, pH 7.2), injected into the right pelvic area.

Clinical observations were conducted over 21 days following each immunization to monitor body weight and overall health. After the challenge, animals were monitored for 30 days for signs such as weight changes, symptoms of tuberculosis (lethargy, disheveled hair), activity, and appetite. After the 30-day period, guinea pigs were euthanized using a guillotine in accordance with ethical standards. Internal organs were examined macroscopically, and bacteriological and histological evaluations were conducted.

### Immunogenicity, immune stability, and protective effectiveness assessment in calves

**Figure 2:**
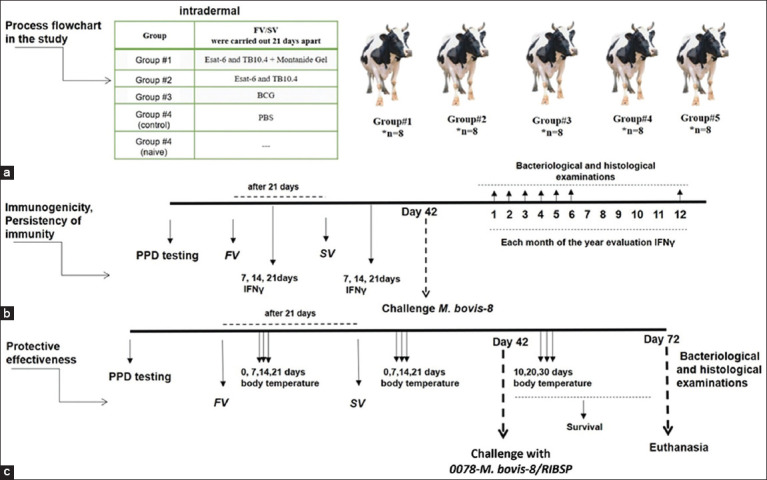
Timeline and sampling schedule for the study of candidate vaccinesin calves (a) Experimental flowchart. (b) Immunogenicity and persistence of the immune response. (c) Protective efficacy. FV = First vaccination, SV = Second vaccination (revaccination).

To evaluate immunogenicity and immune stability, 40 calves were randomly divided into five groups of 8 animals each (n = 8) (Figure 2a). The calves were immunized intradermally with 2 mL (lg 6.25 EID50/mL) of the candidate vaccines on two occasions, 21 days apart (Figure 2b). Blood samples were collected from the jugular vein on days 7, 14, and 21 post-vaccination to measure IFNγ production using enzyme-linked immunosorbent assay (ELISA) (Bovigam, Thermo Fisher Scientific, USA). On day 42 following the first immunization, the animals were challenged with the virulent *M. bovis* strain. Monthly blood samples were collected for 12 months to monitor IFNγ levels. At months 1, 6, and 12, one calf from each group was sacrificed for bacteriological and histological examinations to evaluate the relationship between cellular immunity and protection from infection (Figure 2b).

The protective efficacy of the candidate vaccines was also evaluated. For this, another five groups of calves (n = 8 per group) were immunized with two doses of the vaccine and later challenged with the virulent 0078-*M. bovis*-8/RIBSP strain. The challenge dose was 1 mL containing 1 × 10^6^ CFU per animal, injected into the right pelvic area.

Following the challenge, clinical parameters such as body temperature, activity, appetite, and signs of illness were observed for 30 days (Figure 2c). After this period, the calves were euthanized by exsanguination through severing the jugular vein, carotid artery, and trachea, to avoid interference from chemical euthanasia. Lung tissues were collected and homogenized for bacterial load assessment through culture.

### IFNγ release analysis

To assess IFNγ release in vaccinated calves, heparinized blood was aliquoted at 1.5 mL per well into 96-well plates. Each well contained whole blood and 20 mg/mL of bovine PPD tuberculin (Bovigam, Thermo Fisher Scientific). The plates were incubated for 18–24 h at 37 ± 1°C in a humidified incubator. Plasma was then harvested by centrifugation at 500–770 g for 10–15 min. IFNγ concentrations in the plasma were determined using a commercial ELISA kit (Bovigam TB kit, Thermo Scientific). Absorbance was measured at 450 nm. To calculate optical density indexes (ODIs), the absorbance of PPD-stimulated wells was divided by that of phosphate-buffered saline (PBS)-stimulated controls (typically ~0.1 OD units) (Figure 2b).

### Bacteriology examination

To assess bacterial load, homogenized lung samples were serially diluted and cultured on Lowenstein-Jensen egg medium. The detection limit of this method was 2 × 10^3^ CFU. Mycobacterial burden was expressed in log_10_ CFU per unit lung weight. The organ protection index was calculated by subtracting the CFU log value in immunized animals from that in the control group. A protection index ≥0.5 log was considered indicative of effective mycobacterial inhibition.

### Histology examination

For histological analysis, whole organs, including kidneys, liver, spleen, and lungs, were fixed in 10% neutral-buffered formalin. Sections were prepared and stained using hematoxylin and eosin.

### Electron microscopy

Viral particles of the vector vaccine were concentrated through ultracentrifugation using a Himac CS-150FNX ultracentrifuge (Japan) at 366,000 × *g* for 20 min. The resulting pellet was resuspended in 100 μL of 1× PBS (pH 7.4). Samples were prepared for transmission electron microscopy by adsorption onto copper grids coated with Formvar and reinforced with carbon. Negative staining was done using a 2% aqueous solution of phosphotungstic acid. Visualization was performed using a JEM-100 CX-II transmission electron microscope (JEOL Ltd., Japan) at 80 kV and multiple magnifications. Photographic images were developed and mounted using an Azov photo enlarger [13].

### Evaluation of the genetic insert stability using reverse transcription-polymerase chain reaction (RT-PCR)

To evaluate the stability of the inserted gene, five serial passages of the virus were carried out in embryonated chicken eggs at 34°C. RT-PCR was performed to detect the inserted sequence in the NS gene and compare it to the wild-type virus. RNA was extracted from 100 μL of virus-containing allantoic fluid using the RNeasy kit (Qiagen, Hilden, Germany). RT-PCR was performed using a one-step kit (Ambion, USA) per the manufacturer’s instructions. The primers used were as follows:

- NS-RT-Len (forward): AGCAAAAAGCAGGGTGACAAAG
- PR8-NS1-3UTR (reverse): GAAAACAAGGGTGTTTTTTATTATTAAAT
- PCR was performed in a MiniAmp Thermal Cycler (Applied Biosystems) using the following program: 50°C for 30 min, 95°C for 15 min, followed by 35 cycles of 94°C for 50 s, 54°C for 50 s, and 68°C for 1 min, and a final extension at 68°C for 10 min.

### Statistical analysis

All statistical analyses were performed using GraphPad Prism 8 (GraphPad Software, Inc., La Jolla, CA, USA). Continuous variables were reported as mean ± standard error. One-way analysis of variance (ANOVA) followed by Dunnett’s multiple comparison test was used to compare immune responses, including IFNγ levels, between experimental and control groups. A two-way repeated measures ANOVA with Bonferroni *post hoc* tests was applied to analyze time-course data such as body weight, IFNγ titers, and temperature. Guinea pig survival data were analyzed using the log-rank test. Depending on the data distribution, unpaired t-tests or Mann–Whitney U tests were used to compare outcomes, such as bacterial burden, organ damage scores, and histological changes. Categorical data (e.g., presence of clinical symptoms or lesions) were evaluated using the Chi-square or Fisher’s exact test. Statistical significance was set at p < 0.05.

## RESULTS

### Construction and characterization of vector-based bTB vaccines

**Figure 3:**
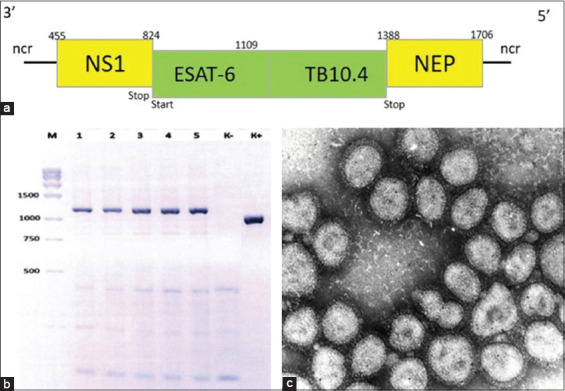
Vaccine strain construction and molecular characteristics. (a) Schematic representation of the recombinant segment of the non-structural protein 1 (*NS1*) gene expressing the ESAT-6 and TB10.4 antigens of the virulent *0078-Mycobacterium bovis-8*/RIBSP strain. The yellow rectangle represents the NS1 regions, while the green rectangles represent the mycobacterial genes. Nucleotide positions are indicated above the rectangles. (b) Agarose gel electrophoresis of reverse transcription-polymerase chain reaction products amplifying the recombinant influenza virus *NS* gene region. Lane M: 100 bp DNA ladder (marker), with fragment sizes indicated in base pairs; Lanes 1–5: Recombinant virus samples from different serial passages; Lane K− = Negative control (no template); Lane K+ = Positive control (wild-type *NS1* gene). (c) Electronic microimage of recombinant vaccine strain virions.

We produced a vaccine strain expressing the *M. bovis* mycobacterial proteins ESAT-6 and TB10.4 from the NS1 open reading frame of the avian influenza virus through reverse genetics with virus replication in embryonated chicken eggs. The virus was attenuated by modifying the NS1 protein by inserting alien sequences derived from the target protein amino acid region 124 (Figure 3a).

No significant differences in the tertiary structure of the initial recombinant influenza virus strains expressing mycobacterial antigens versus their passaged variants were identified in the homology models. The first cloning stage showed a preserved genetic chimeric structure in the NS1 genome segment, as confirmed by RT-PCR (Figure 3b). As demonstrated by electronic microimagery, the virion morphology of the recombinant viruses corresponded to that of the avian influenza viruses. The virions were spherical and enclosed in a bilayer supercapsid with ~10 nm glycoprotein spikes, which determine the hemagglutinative or neuraminidase activity (Figure 3c).

The recombinant strain was restored in embryonated chicken eggs. A higher virus accumulation rate was observed in 10-day-old chicken eggs incubated at 37°C ± 0.5°C. The recombinant vector expressing the mycobacterial antigens showed a high hemagglutination activity of 1:128 at an infectious activity level of lg 6.75 ± 0.07 EID50/0.2 mL. Experiments at 26°C and 34°C ± 0.5°C produced negative results as virus growth was practically absent.

### Safety assessment in guinea pigs

The first phase of the study was to assess the safety of the candidate vaccines (Figure 1). Innocuity testing did not identify any clinical signs in any of the guinea pig groups for 10 days after a single subcutaneous injection. Examination of the skin and visible mucosa did not reveal any changes. The muscular tone in the experimental animals was moderate. Motor activity was as usual, with no deviations from the physiological norm or motor coordination impairment. No signs of inflammation were observed at the injection site. However, several animals had local reactions in the form of swelling, which completely resolved within 3 days of injection (Table 1).

**Table 1:** Safety (innocuity) testing results in guinea pigs over a 10-day observation period. The numbers in each cell indicate the number of surviving animals per day (bottom row) and the number of observed deaths (top row, bold). “Local reaction” and “Tissue reaction at the injection site” columns indicate visual assessment of inflammation, swelling, or necrosis.

Groups	Survival (days of observation)										The local reaction	Tissue reaction at the site of injection

	1	2	3	4	5	6	7	8	9	10		
Group #1	0	0	0	0	0	0	0	0	0	0	- - -	- - -
	8	8	8	8	8	8	8	8	8	8		
Group #2	0	0	0	0	0	0	0	0	0	0	- - -	- - -
	8	8	8	8	8	8	8	8	8	8		
Group #3	0	0	0	0	0	0	0	0	0	0	- - -	- - -
	8	8	8	8	8	8	8	8	8	8		
Group #4	0	0	0	0	0	0	0	0	0	0	- - -	- - -
	8	8	8	8	8	8	8	8	8	8		

1) Numerator: Number of dead animals; 2) Denominator: Number of surviving animals; 3) “+”: insignificant reaction (single discrete proliferation of white fibrous connective tissue 2.0–2.5 mm in diameter; 4) “- - -”: No observable reaction

**Figure 4:**
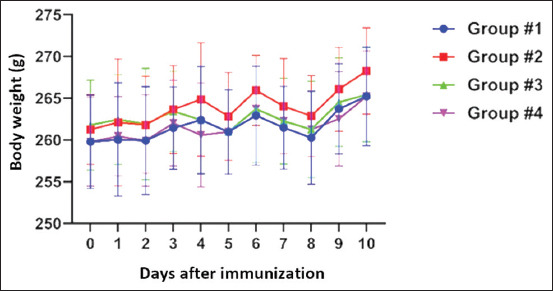
Body weight dynamics of guinea pigs after the first immunization. The observation period was 10 days. The standard deviations for the mean body weight values of the groups are presented as error bars.

The data in Table 1 indicate that introducing the tested tuberculosis vaccines or saline solution did not cause death in guinea pigs; the experimental animals stayed clinically healthy and had no local or tissue reaction at the injection site for the entire 10 days of observation. The body weight of the experimental group animals was not different from that of the control group. Animals in all groups exhibited a growth in body weight, as shown in Figure 4.

### Assessment of protective effectiveness in guinea pigs

**Figure 5:**
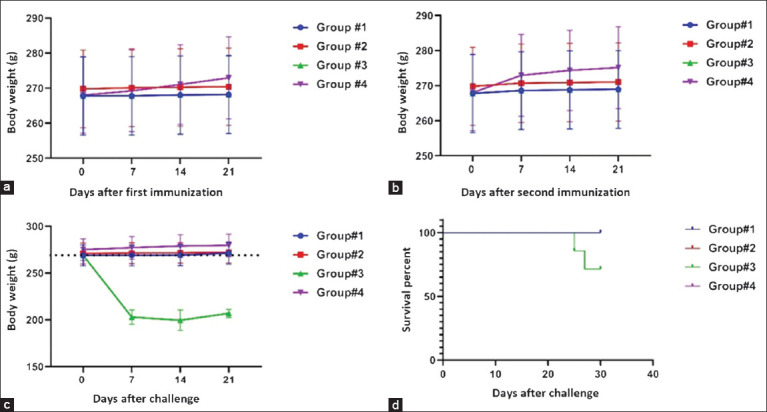
Body weight dynamics of Guinea pigs over time, the standard deviations for the mean percent deviation of the body weight values of the groups are presented as error bars. Survival curves were analyzed for the experimental and control groups using the log-rank test with p = 0.006. (a) Body weight in grams (mean value) after the first immunization. (b) Body weight in grams (mean value) after the second immunization. (c) Body weight in grams (mean value) after challenge with the virulent strain. (d) Survival curves of challenged guinea pigs.

The next phase of the study was to assess the protective effectiveness of the candidate vaccines in guinea pigs. The rates of body weight growth in the experimental group on the 6^th^ and 7^th^ days after the first and second doses of the vaccines were comparable to those in the control group. The body weight of the experimental group animals increased significantly toward the final days of the experiment. These data are of considerable interest because the tested candidate vaccines did not produce a negative impact on the body weight of experimental guinea pigs (Figures 5a and b).

Figure 5 presents the survival data of guinea pigs challenged with 0078-*M. bovis*-8/RIBSP. A statistical analysis of survival curves for guinea pigs challenged with a virulent 0078-*M. bovis*-8/RIBSP strain showed a high significance of the differences (log-rank test, p = 0.006). Comparison of death curves for challenged guinea pigs exhibits a positive effect of the candidate vaccines on the life expectancy of guinea pigs. On days 25 and 27 after the challenge, two animals in the control group died, whereas all animals in the experimental (Group #1 and Group #2) and naive (Group #4) groups were alive.

Challenged animals from the control group (Group #3 - Saline) had enlarged lymphatic nodes at the injection site, and two had developed unhealing lesions (Figure 5d). These two animals died. The mortality rate in this group was 30% (n = 8). Necropsy of the animals revealed internal organ changes caused by tuberculosis infection. Their lungs, livers, and spleens had grayish-yellow lumps on their surfaces, some of which merged with each other. The right inguinal lymph nodes were enlarged and contained creamy pus.

All guinea pigs were euthanized on day 30 after the challenge for macroscopy and histology examinations. The severity of experimental tuberculosis was expressed using the organ damage index. The macroscopy results were characterized by the absence of specific inflammation in the collateral inguinal lymph nodes and lesser damage to the lungs and spleen (Table 2).

**Table 2:** Macroscopic organ damage index in guinea pigs (n = 8 per group). The index was assessed based on visual scoring of gross pathological lesions in the lungs and spleen after challenge. Values represent mean ± standard deviation.

Groups	Organ damage index (n = 8)		Total organ damage index

	Lungs	Spleen	
Group #1	0.6 ± 0.3	0.25 ± 0.12	0.85
Group #2	0.5 ± 0.2	0.25 ± 0.12	0.75
Group #3	3.75 ± 0.12	3.7 5 ± 0.12	7.5
Group #4	---	---	---

Table 2 shows that the macroscopy results in the experimental groups were characterized by the absence of specific inflammation foci in the lungs (0.5–0.6 vs. 3.75) and minimal damage in the spleen (0.25 vs. 3.75). By total index, vaccinated animals showed significantly lower lesion scores in the lungs and spleen (0.85 and 0.75 total index in Groups #1 and #2, respectively), compared with the control group (7.5 total index). Animals in Group #4 had no changes in their organs as they were not subjected to any manipulations

**Figure 6:**
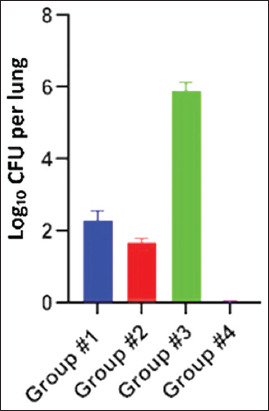
Isolation of mycobacteria from the lungs of guinea pigs challenged with *0078-Mycobacterium bovis-8*/RIBSP Results are expressed as Log_10_ of CFU per lung. Mean ± SD is shown for each group. SD = Standard deviation.

In an assessment of lung cultures, all immunized animals exhibited significantly lower mycobacterial growth than those in the control group (Group 3) (Figure 6).

**Figure 7:**
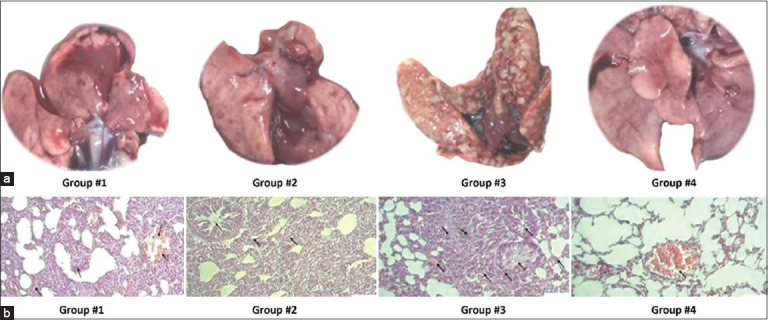
Macroscopic and histopathological assessment of lung tissue 30 days after challenge with *0078-Mycobacterium bovis-8*/RIBSP. (a) Representative macroscopic images of lungs from the experimental groups. (b) Macroscopic images of lungs from the experimental groups. (b) Histopathological analysis of hematoxylin and eosin-stained lung tissue at 400× magnification. Arrows indicate pathological changes. Groups: Group 1, vaccine with adjuvant; Group 2, vaccine without adjuvant; Group 3, control (challenged, saline); Group 4, naive control.

Histological analysis revealed various morphological changes in guinea pig lung tissues across different groups. In Group #1, the lung tissue maintained its structure; however, thickening of the interalveolar septa was observed in the presence of macrophages. These macrophages appeared as large mononuclear and multinucleate cells. Pronounced vascular congestion was also noted. The observed morphological changes were focal in nature (Figure 7). In Group #2, the interalveolar septa of the lungs thickened due to cell proliferation, leading to narrowing of the bronchial lumen due to spasms, accompanied by edema around the bronchi. Microscopically, the cellular composition of the interalveolar septa comprised lymphocytes, large mononuclear cells, and occasional erythrocytes. Additionally, signs of edema were observed in the alveolar septal wall (Figure 7). In Group #3, morphological changes in the lung tissues were characterized by productive interstitial inflammation with focal micronecrosis accompanied by vascular congestion. The bronchial epithelium exhibited desquamation and edema around the bronchi. In Group #4, the lung structure remained preserved, with vascular congestion (Figure 7).

Group #1 exhibited a specific decrease in *M. bovis* growth (p < 0.05); however, the protection index was relatively low (+0.2 lg). Group #2 exhibited a significant reduction in *M. bovis* isolation (p < 0.01) and a comparatively higher protection index (+0.60 lg). These findings demonstrate the prophylactic effect of candidate vaccines. As shown in Figure 6, Group #3 exhibited *M. bovis* growth in their lungs and had no protection index.

### Immunogenicity and immune stability assessment

**Figure 8:**
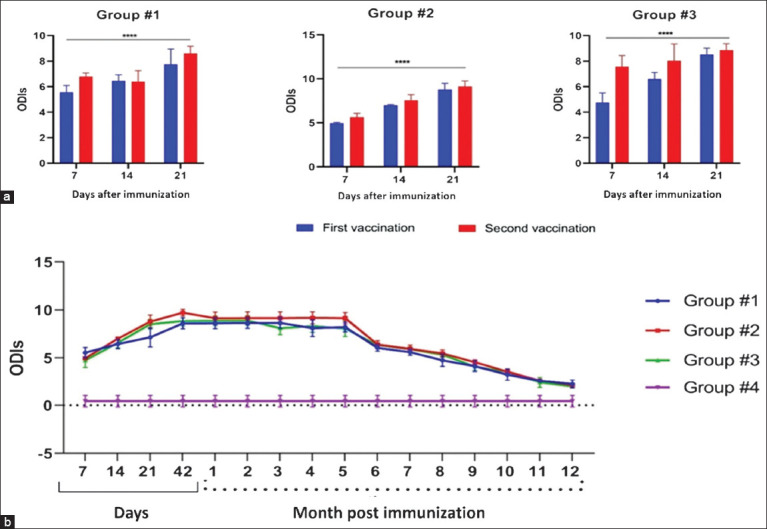
Interferon-gamma (IFNγ) production over time. Analysis of IFNγ release in enzyme-linked immunosorbent assay, where the ODI results for animals stimulated with PPD were divided by the results for cultures derived from animals stimulated with saline. Absorption in the control and test samples was measured at 450 nm. We used approximately 0.1 units of OD for normalizing individual measurements for the saline controls (Group #4). (a) Assessment of immune response after double immunization of calves. (b) Intensity of post-vaccination immunity in calves for 12 months.

We performed a comparative assessment of T-cell immunity in the study groups after immunizing calves twice with our candidate vaccines and a commercial BCG vaccine. We discovered considerable differences between the experimental groups and an increase in IFNγ cells’ production. An analysis of IFNγ production in Dunnett’s Multiple Comparisons test at a confidence interval of 95% demonstrated statistically significant differences (p < 0.0001) in each group on days 7, 14, and 21 after each immunization, as shown in Figure 8a. In the negative control group, animals that were immunized with saline, we found no IFNγ cells with ELISA.

Our assessment of the immunogenic properties of the studied vaccine samples following two subcutaneous immunizations showed that the studied formulations and the BCG vaccine elicited persistent immunity in calves irrespective of the antigen and adjuvant (Al + 3) content (Figure 8).

The intensity of post-vaccination immune response in calves was different between the groups immunized with the candidate vaccines and the control substance-immunized group, i.e., BCG (Figure 8). As shown in Figure 8b, the *M. bovis* antigens under study demonstrated differences in their immunogenic characteristics (IFNγ production), which can be attributed to the effect of aluminum hydroxide. This specific vaccine adjuvant catalyzes immunogenicity and can hinder/inhibit the immune response in calves. The adjuvant-free formulation produced the highest titers of IFNγ in month 5, whereas the adjuvant-containing vaccine produced comparatively lower IFNγ titers. Overall, both candidate vaccines enhanced the immune response and its persistence in a manner that is not inferior to that of the BCG vaccine (Group #3).

We demonstrated that the high titers of IFNγ cells peaking at 2–4 weeks following vaccination provide effective protection against challenge, as evidenced by our histology results.

### Calves immunization/challenge studies

**Figure 9:**
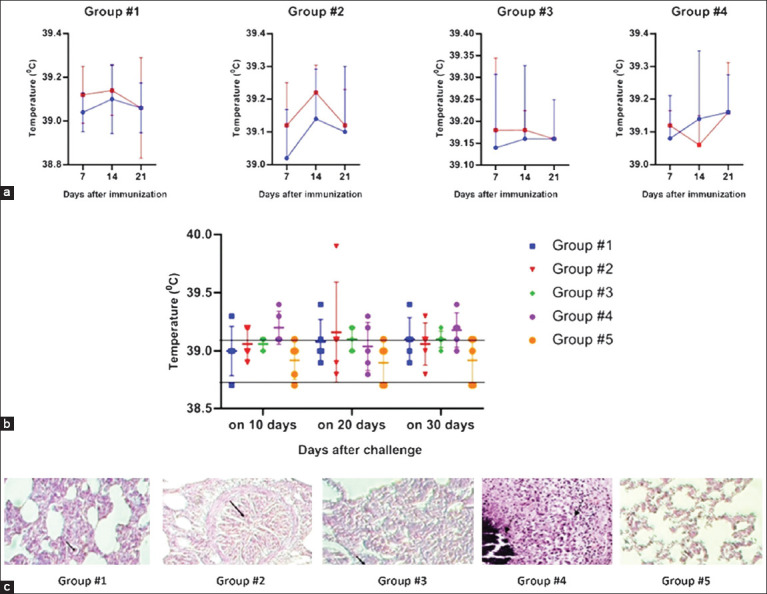
Analysis of body temperature and histopathological lung changes in calves over time (a) Body temperature fluctuations in calves after two immunizations (blue and red lines indicate the first and second vaccinations, respectively). The observation period was 0, 7, 14, and 21 days after each vaccination. The standard deviations (SD) for mean body temperature values in groups are provided as error bars. (b) Fluctuations in body temperature in calves after challenge. The observation period was 10, 20, and 30 days after the challenge; the SD for mean body temperature values in groups is provided as error bars. (c) Histological structure of the lungs (hematoxylin and eosin 200×). The arrows indicate representative changes.

The general state of health of calves after each immunization with the studied vaccines and BCG was normal, similar to that in Groups 4 and 5. The body temperature of calves on days 0, 7, 14, and 21 after the first and second immunization was normal in all groups without any significant fluctuations (Figure 9a). In an IFNγ release assay using ELISA to calculate optical density indices (ODI), we used Dunnett’s multiple comparisons test at a confidence interval of 95%. Significant differences (p < 0.0001) were observed between the first and second immunizations.

After euthanizing the calves, we visually examined the injection sites and found no specific inflammation foci. We necropsied the animals and assessed the macroscopic changes in the organs (lymph nodes, liver, spleen, and lungs). The severity of bTB infection was expressed using the Organ Damage Index. Table 3 presents the internal organ macroscopic damage indices.

**Table 3:** The internal organ macroscopic damage index.

Groups	Organ damage index (n = 8)					Total organ damage index

	Lungs	Spleen	Liver	Inguinal LNs		

				Right	Left	
Group #1	0.75 ± 0.3	0.25 ± 0.12	0.5 ± 0.3	0.22 ± 0.12	0.21 ± 0.12	1.93 ± 0.3
Group #2	0.5 ± 0.2	0.25 ± 0.12	0.6 ± 0.3	0.25 ± 0.12	0.25 ± 0.12	1.85 ± 0.17
Group #3	0.65 ± 0.2	0.25 ± 0.10	0.5 ± 0.0	0.22 ± 0.12	0.21 ± 0.00	1.83 ± 0.3
Group #4	1.4 ± 0.5	0.5 ± 0.5	0.5 ± 0.3	0.20 ± 0.2	0.21 ± 0.2	2.81 ± 0.3
Group #5	---	---	---	---	---	---

Values represent the mean ± standard deviation of the macroscopic lesion scores in the right and left lung, spleen, liver, and inguinal lymph nodes. The organ damage index was based on visual scoring criteria post-challenge with *Mycobacterium bovis*. The total organ damage index was calculated as the sum of individual organ scores

Visual examination of calves’ organs (lungs, liver, spleen, and inguinal lymph nodes) for specific damage after two immunizations with the studied vaccines showed a decrease in the total organ damage index in all of the immunized animals. Calves in Group #5 did not have any macroscopic change in their organs, as they were not subjected to any manipulations.

Macroscopic scoring revealed that the total organ damage index was 1.93 ± 0.3 in Group #1, 1.85 ± 0.17 in Group #2, and 1.83 ± 0.3 in the BCG-vaccinated Group #3, whereas the unvaccinated control animals in Group #4 exhibited a significantly higher index of 2.81 ± 0.31 (Table 3). These findings confirm the protective effect of the tested vaccines against *M. bovis*-associated tissue damage.

The body temperature of vaccinated calves developed after being challenged was within the norm (Figure 9b); however, the introduction of the virulent *M. bovis* strain led to different body temperature changes in the various animal groups. A 0.2–0.3°C rise in the experimental groups (Groups 1, 2, and 3) and a 0.2–0.7°C rise in the control group (Group 4) were observed only from day 10 to day 30 after the challenge. The mean body temperature value was measured 3 days before the challenge as the baseline.

In Group #1, the temperature changes were relatively small and limited to a narrow range. This suggests that the vaccine could offer a certain degree of protection from the virulent strain, as the body temperature of the calves remained relatively stable. In Group #2, the body temperature changes were larger than those in Group #1. However, the temperatures remained relatively stable, indicating a certain level of immune response and protection. Changes in temperature were minimal in Group #3 as the temperature remained relatively stable throughout the observation period. This suggests that the BCG vaccine could provide effective protection against the virulent strain.

Temperature changes were more significant in Group #4 than in any vaccinated group. Calves exhibited elevated body temperature, especially on days 10 and 20 after the challenge. This suggests that the absence of immunization led to the absence of protection, causing a more intense reaction to the virulent strain.

### Histopathological and microbiological evaluation in calves

Analysis of the histological structure of lung tissue in calves, Group #1 (Figure 9c) showed that the structure of the lungs is wholly preserved; foci of interstitial productive inflammation with reparative processes in the form of thin small vessels are noted. In Group #2, the structure of the lung tissue is preserved, the interalveolar septa of the lung are not thickened, the lumen of the alveoli is free, the bronchial wall is thickened due to edema, and the lumen is sharply narrowed due to spasm (Figure 9c). Morphological changes in the lungs of calves in Group #3 were characterized by productive interstitial inflammation, which was focal; granulation tissue with small vessels began to form in some areas. The interalveolar septum thickened in other parts of the lung due to productive inflammation with macrophages and newly formed thin vessels (Figure 9c). The histological structure of the lungs is preserved in naive calves in Group 4. The bronchi walls are not thickened, and the epithelial cells are preserved. The vessels’ walls are thin, with single erythrocytes in the lumen. The interalveolar septa are thin, the vessels are collapsed, and the alveolar lumen is free (Figure 9c).

Analyzing the data obtained from microscopic studies, we can conclude that the infection of animals with the virulent strain 0078*-M. bovis*-8/RIBSP immunized with a vaccine candidate containing 15% Montanide Gel adjuvant and the commercial BCG vaccine led to minor morphological changes in the lung. Reproducing the tuberculosis model with the introduction of a virulent strain, the following morphological changes were observed in the lungs. Productive focal interstitial inflammation developed in the lung tissue. In addition, reparative processes with newly formed small vessels were noted (Figures 8a and c).

Analysis of the morphological changes in the lungs of calves immunized with the vaccine candidate without an adjuvant showed that the histological structure of the lung was not disturbed. Only minor changes that did not affect the general condition of the animals and were not infectious were detected.

In Group 4, characteristic morphological changes for tuberculosis in the lungs were detected, including multiple foci of necrosis. Caseous necrosis revealed remnants of nuclei and was surrounded by a granulomatous inflammatory reaction containing lymphocytes, epithelioid cells, macrophages, and single Langhans cells (Figure 9c). The lung structure of calves in Group #5 had a typical histological structure, as calves remained clean without any manipulation (Figure 9c).

**Figure 10:**
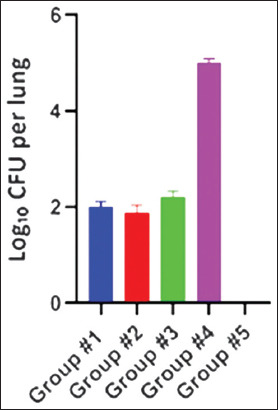
Isolation of mycobacteria from the lungs of calves challenged with *0078-Mycobacterium bovis-8*/RIBSP.

A culture test of the lungs of euthanized calves challenged with a virulent 0078-*M. bovis*-8/RIBSP strain (30 days after vaccination) showed a significantly lower growth of *M. bovis* in all vaccinated groups compared with the control group (Group #4, Figure 10).

Immunizing calves with the studied candidate vaccines (1.83–1.93 lg CFU) and the BCG vaccine provided the animals with effective protection, which the control group calves immunized with saline did not have (5.8 lg CFU).

Thus, vector-based candidate vaccines for bTB developed at the RIBSP were found to be innocuous for guinea pigs and calves and offer protection against a virulent 0078-*M. bovis*-8/RIBSP strain. These vaccines were highly immunogenic and elicited intense immunity lasting over 12 months in calves immunized twice.

## DISCUSSION

### Tuberculosis as a persistent zoonotic threat

Tuberculosis holds a unique position among infectious diseases. It affects humans, all animal species, and birds. Despite over 100 years since its causative agent was discovered by Robert Koch in 1882, the disease remains widespread. The most severe consequence of TB in animals is the potential for zoonotic transmission to humans, primarily through the consumption of contaminated animal products or contact with infected animals. Controlling TB in animals is a critical priority worldwide, with significant implications for public health and economic stability. bTB, a zoonotic disease caused by *M. bovis*, is transmitted to humans through the consumption of raw milk, aerosols, or dust containing the causative agent [14].

### Limitations of current vaccination strategies

Vaccination is a key tool in the fight against bTB. Prophylactic measures for bTB largely depend on the traditional BCG vaccine. Despite variations in its protective efficacy across species, BCG remains the only commercially available vaccine for mitigating the risk of large-scale culling and reducing the spread of bTB among cattle. However, BCG vaccination has limitations, including the inability to differentiate vaccinated animals from infected ones in tuberculin skin tests. In addition, the BCG strain lacks the RD1 genomic region encoding the virulent antigens ESAT-6 and CFP-10, which impairs the development of immunologic memory for these antigens [14, 15].

### Advances in viral vector platforms

Viral vectors are an advanced method for delivering and presenting antigens to the immune system. Their suitability is determined by criteria such as safety, absence of pre-existing immunity, and the ability to overcome post-vaccination vector-specific immunity. These factors are essential for eliciting an effective immune response to the target antigen [16].

### Advantages of influenza virus-based vectors

Influenza virus-based vector vaccines offer a promising alternative to inactivated vaccines because they enable native expression and efficient delivery of antigens to immunologically active sites. The production of new vector vaccines using influenza viruses is also rapid and scalable [16, 17]. Recombinant avian influenza A viruses expressing *M. tuberculosis* antigens, such as ESAT-6 and Ag85a, are safe and induce robust % T-cell responses comparable to those induced by the BCG vaccine [18, 19]. Furthermore, a recombinant influenza vector expressing *M. bovis* antigens ESAT-6 and TB10.4 has demonstrated effective protection against *M. bovis* in mouse models [10].

### Development of a novel influenza-based candidate vaccine

Several factors influence the protective efficacy of the BCG vaccine against *M. bovis*, including the vaccine strain, dosage, and route of administration [20, 21]. In this study, we used reverse genetics technology to develop influenza vector-based candidate vaccines expressing ESAT-6 and TB10.4 antigens derived from the virulent 0078-*M. bovis*-8/RIBSP strain.

### Role of the NS1 protein in antigen delivery

The influenza virus’s non-structural protein NS1 was selected for antigen insertion due to its capacity to accommodate foreign sequences while preserving viral viability and immunogenicity. NS1 is abundantly expressed in infected cells and elicits strong CD8+ T-cell responses. Genetic modification of the NS1 protein results in an attenuated phenotype while maintaining its immunostimulatory properties [22, 23].

### Vaccine formulation and antigen selection

The candidate vaccine was developed as a lyophilized powder for subcutaneous administration. The primary antigens, ESAT-6 and TB10.4, are well-characterized immunodominant proteins involved in *M. bovis* virulence and host immune modulation. Their role in activating CD4+ and CD8+ T-cells makes them ideal targets for inducing protective immunity [24–28].

### Safety and immunogenicity outcomes

Our study demonstrated that both candidate vaccines were safe, causing no adverse effects such as behavioral changes, weight loss, or mortality in vaccinated animals. Histological examination revealed minimal internal organ alterations, particularly in animals immunized with the adjuvant-free formulation, which showed no signs of inflammation or pathology.

### Immune response and duration of protection

Immunogenicity analysis using IFNγ ELISA revealed a robust response that peaked on day 42 and persisted for up to 12 months. Statistically significant differences in IFNγ production between the experimental and control groups were recorded from day 21 to month 5 (p ≤ 0.000001). The non-adjuvanted vaccine demonstrated higher IFNγ levels than the adjuvant version and BCG.

### Histopathological confirmation of efficacy

Histological assessment of post-challenge lung tissues confirmed that calves vaccinated with the adjuvant-free formulation displayed minimal structural damage. This was in contrast to BCG- and adjuvanted vaccine groups, which exhibited mild lesions, and unvaccinated controls, which showed severe pathology. These findings suggest that the non-adjuvanted formulation has superior protective efficacy.

### Consistency with previous findings and study limitations

Our results align with previous studies which have shown that BCG vaccination reduces granulomatous lesions in cattle [29, 30], white-tailed deer [31], and mice [32]. A limitation of this study is the absence of Western blot analysis to verify antigen expression; however, this will be addressed in future research. Further investigation of immune cell subpopulations and antigen-specific responses is warranted. Although the viral titer was standardized using EID_50_ in embryonated chicken eggs (ECEs), the quantity of expressed ESAT-6 and TB10.4 antigens per dose was not quantified in this study. This limitation will also be addressed in future research using direct antigen quantification methods.

### Practical considerations and DIVA strategy potential

Immunogenicity and protective efficacy demonstrated by IFNγ production and histological outcomes underscore the potential of the candidate vaccines. The influenza vector system offers a scalable and adaptable platform from a practical standpoint. The production of embryonated chicken eggs is cost-effective, and the freeze-dried form ensures stability and ease of transport. Regulatory challenges and differentiation between vaccinated and infected animals (DIVA strategy) remain key considerations for large-scale implementation.

Thus, the candidate influenza vector-based vaccines encoding ESAT-6 and TB10.4 demonstrated robust immunogenicity, long-lasting protection, and safety in guinea pigs and calves. These results support their potential use in bTB control programs in Kazakhstan and internationally.

## CONCLUSION

This study successfully developed and evaluated two candidate influenza vector-based vaccines encoding *M. bovis* ESAT-6 and TB10.4 antigens, demonstrating robust immunogenicity, protective efficacy, and safety in guinea pigs and calves. The recombinant vaccines, particularly the adjuvant-free formulation, induced strong IFNγ responses that peaked at day 42 and persisted for up to 12 months, surpassing even the BCG vaccine in immunogenicity. Histopathological analysis confirmed minimal tissue damage in animals immunized with the adjuvant-free candidate, supporting its superior protective efficacy.

The use of influenza viral vectors presents a scalable and cost-effective alternative to conventional BCG vaccines. The lyophilized vaccine form enhances stability and simplifies storage and distribution, making it especially suitable for use in endemic or resource-limited settings. The NS1 protein insertion strategy allows effective antigen presentation without compromising viral replication, and the use of embryonated chicken eggs facilitates rapid production.

Key strengths of this study include the demonstration of long-lasting cell-mediated immunity, confirmation of cross-species effectiveness using both guinea pig and calf models, and histological and bacteriological evidence of reduced pathology and bacterial load. The candidate vaccines elicited greater IFNγ responses than BCG and were well tolerated, with no adverse clinical signs. Additionally, the study validated the influenza NS1 protein as a stable and effective antigen delivery platform.

However, the study had some limitations. The amount of expressed ESAT-6 and TB10.4 antigens per dose was not quantified, and western blot validation of antigen expression was not performed. Furthermore, antigen-specific memory T-cell responses and immune cell subset dynamics were not assessed. Finally, the results are limited to controlled experimental conditions and have not yet been validated under natural field exposure.

Future research should focus on quantifying antigen expression per dose, characterizing immune cell populations and memory responses, assessing DIVA compatibility, and conducting large-scale field trials in naturally exposed livestock. It would also be valuable to explore alternative delivery routes, such as mucosal immunization, to enhance practical application.

In conclusion, the candidate influenza vector-based vaccines demonstrated excellent safety, strong and durable immune responses, and protective efficacy against *M. bovis*. These findings support their potential role in national and international bTB control strategies, offering a promising and scalable alternative to existing vaccination approaches.

## AUTHORS’ CONTRIBUTIONS

AN and MK: Conceptualization and drafted and revised the manuscript. BY and KS: Conceptualization and methodology. KZ: Conceptualization. NS: Methodology. SS: Investigation. KJ, RA, SK, AI, YS, EK, ZA, SM, and MS: Investigation. ZhA: Investigation and edited the manuscript. HSY, SN, AK, and BK: Study design and supervision. All authors have read and approved the final manuscript.
